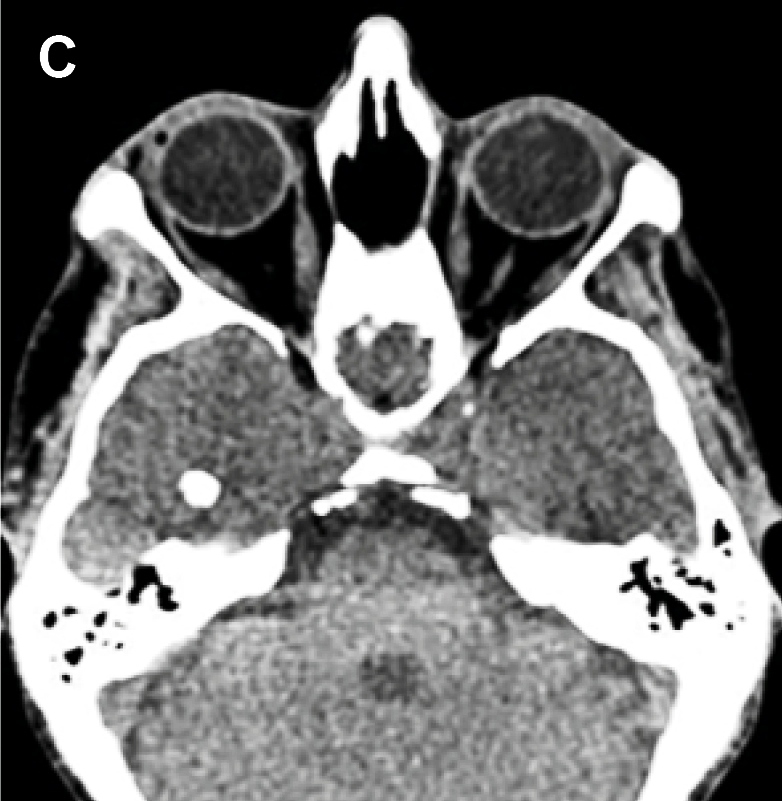# Reactivation of neurocysticercosis: calcified nodular lesion and perilesional edema

**DOI:** 10.1590/0037-8682-0560-2021

**Published:** 2022-02-25

**Authors:** Larissa Grazielle Souza Ribeiro, Helen Cristina Marcusso, Lucas Giansante Abud

**Affiliations:** 1 Hospital São Francisco, Documenta Centro Avançado de Diagnóstico por Imagem, Ribeirão Preto, SP, Brasil.; 2 Hospital São Lucas, Medicina Diagnóstica, Ribeirão Preto, SP, Brasil.

A 53-year-old man presented to the emergency department with headache and seizures. Axial fluid-attenuated inversion recovery (FLAIR) magnetic resonance imaging (MRI) revealed a nodular focus in the right temporal lobe with a halo of T2 hyperintensity (Figure A). Computed tomography (CT) confirmed a calcified image with edema (Figure B). The patient's symptoms improved with clinical treatment, and CT after 7 months showed maintenance of cerebral calcification with the disappearance of edema, suggesting total regression of the inflammation (Figure C). Neurocysticercosis (NCC) develops after the ingestion of *Taenia solium* eggs and represents the parasitic disease with the greatest tropism for the human central nervous system^1^. It is the most prevalent cause of acquired epilepsy and is a major public health problem worldwide^1^ ^,^ ^2^. Calcifications, which represent the final stage, are the most common radiological findings among NCC cases. The presence of perilesional edema around a calcification supports the diagnosis of NCC reactivation, which can lead to headaches, seizures, and disabling epilepsy^2^ ^,^ ^3^. Some hypotheses can explain the reactivation of the disease. For example, perilesional edema may have resulted from the death of parasites that were still viable and incompletely calcified^2^ ^,^ ^3^. Another possibility is the release of antigens from dead cysticerci, which, for unclear reasons, can be recognized by the host, triggering an inflammatory response^2^. Regardless of the explanation, clinicians and radiologists should know that patients with calcified lesions may experience NCC reactivation^2^. Thus, this diagnosis should be made using imaging findings, and prompt care support must be provided to avoid unfavorable outcomes. 

**Figure:**
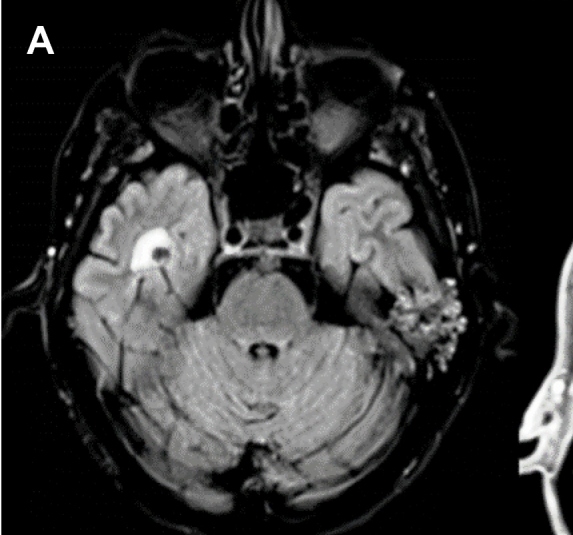

**Figure:**
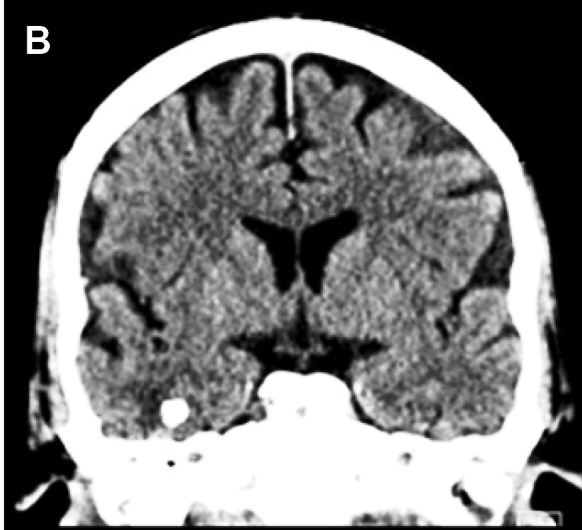

**Figure:**